# PBPK Modeling as a Tool for Predicting and Understanding Intestinal Metabolism of Uridine 5′-Diphospho-glucuronosyltransferase Substrates

**DOI:** 10.3390/pharmaceutics13091325

**Published:** 2021-08-24

**Authors:** Micaela B. Reddy, Michael B. Bolger, Grace Fraczkiewicz, Laurence Del Frari, Laibin Luo, Viera Lukacova, Amitava Mitra, Joyce S. Macwan, Jim M. Mullin, Neil Parrott, Aki T. Heikkinen

**Affiliations:** 1 Early Clinical Development, Department of Clinical Pharmacology Oncology, Pfizer, Boulder, CO 80301, USA; 2 Simulations Plus Inc., Lancaster, CA 93534, USA; bolger@simulations-plus.com (M.B.B.); grace@simulations-plus.com (G.F.); viera@simulations-plus.com (V.L.); joyce@simulations-plus.com (J.S.M.); jim@simulations-plus.com (J.M.M.); 3 PKPD Department, Pierre Fabre Laboratories, 31100 Toulouse, France; laurence.del.frari@pierre-fabre.com; 4 Material & Analytical Sciences, Boehringer Ingelheim Pharmaceuticals, Inc., Ridgefield, CT 06877, USA; laibin_2.luo@boehringer-ingelheim.com; 5 Clinical Pharmacology and Pharmacometrics, Janssen Research & Development, Springhouse, PA 19477, USA; amitra24@its.jnj.com; 6 Pharmaceutical Sciences, Roche Pharmaceutical Research and Early Development, Roche Innovation Center Basel, F. Hoffmann-La Roche, 4070 Basel, Switzerland; neil_john.parrott@roche.com; 7 Admescope Ltd., Fi-90620 Oulu, Finland; aki.heikkinen@admescope.com

**Keywords:** GastroPlus, PBPK, UGT, intestinal metabolism, gut extraction, absorption modeling, oral bioavailability, IVIVE, phase II metabolism

## Abstract

Uridine 5′-diphospho-glucuronosyltransferases (UGTs) are expressed in the small intestines, but prediction of first-pass extraction from the related metabolism is not well studied. This work assesses physiologically based pharmacokinetic (PBPK) modeling as a tool for predicting intestinal metabolism due to UGTs in the human gastrointestinal tract. Available data for intestinal UGT expression levels and in vitro approaches that can be used to predict intestinal metabolism of UGT substrates are reviewed. Human PBPK models for UGT substrates with varying extents of UGT-mediated intestinal metabolism (lorazepam, oxazepam, naloxone, zidovudine, cabotegravir, raltegravir, and dolutegravir) have demonstrated utility for predicting the extent of intestinal metabolism. Drug–drug interactions (DDIs) of UGT1A1 substrates dolutegravir and raltegravir with UGT1A1 inhibitor atazanavir have been simulated, and the role of intestinal metabolism in these clinical DDIs examined. Utility of an in silico tool for predicting substrate specificity for UGTs is discussed. Improved in vitro tools to study metabolism for UGT compounds, such as coculture models for low clearance compounds and better understanding of optimal conditions for in vitro studies, may provide an opportunity for improved in vitro–in vivo extrapolation (IVIVE) and prospective predictions. PBPK modeling shows promise as a useful tool for predicting intestinal metabolism for UGT substrates.

## 1. Introduction

Physiologically based pharmacokinetic (PBPK) modeling has become a valuable tool for understanding drug pharmacokinetic (PK) properties in humans based on nonclinical and in vitro data prior to the first in-human study [1,2]. Additionally, PBPK modeling is an important tool for predicting first-pass loss from intestinal metabolism for cytochrome P450 (CYP) enzymes in humans [3,4] and nonclinical species (e.g., the beagle dog [5]). Extensive literature illustrates the ability to predict the fraction of drug escaping first-pass intestinal metabolism, Fg, for CYP3A substrates (e.g., [4,6,7]). Other enzymes besides CYPs are expressed in the gastrointestinal (GI) tract but are less well studied. Multiple uridine 5′-diphospho-glucuronosyltransferases, UGTs, are expressed in the GI tract, including UGT1A1, 1A3, 1A4, 1A7, 1A8, 1A10, 2B7, 2B15, and 2B17 [8,9,10,11]. This paper builds on the strong foundation of predicting intestinal metabolism for CYP substrates but expands to this less-studied area, the UGTs.

Several first-pass processes can potentially reduce exposure before the drug reaches systemic circulation. PBPK models incorporate system-specific parameters such as enzyme expression levels in the GI tract and liver, as well as compound-specific parameters such as enzyme maximum rate of metabolism, Vmax, and Michaelis constant, K_m_, and protein binding data, to predict PK properties such as Fg. Oral administration is subject to first-pass losses from incomplete absorption (i.e., fraction of dose absorbed, Fa, <1), first-pass intestinal metabolism (i.e., Fg < 1) and first-pass hepatic metabolism (i.e., fraction moving from the portal vein through the liver—hepatic availability, Fh, <1). The loss of bioavailability, F, due to first-pass processes can be estimated as:

F = Fa × Fg × Fh.(1)

Hepatic metabolism is well-recognized as a main contributor affecting oral bioavailability. However, intestinal metabolism can significantly impact bioavailability, and, in some cases, potentially more than hepatic first-pass metabolism due to less binding in the small intestines (e.g., in PBPK model development for predicting intestinal metabolism for CYP3A4 substrates, setting the unbound fraction in the gut to 1 resulted in more accurate predictions than other assumptions, such as the unbound fraction in plasma, which, in most cases, resulted in a significant underestimation of intestinal metabolism [4]) and differences in expression levels in the intestines compared to the liver [12]. Additionally, the process of transcellular passive absorption exposes all molecules to metabolic enzymes in the intestine, whereas only molecules absorbed into the hepatocyte are exposed to liver enzymes. The complexity of PK processes involved with glucuronidation, e.g., the importance of transporters for glucuronide conjugates as well as enterohepatic cycling involving these glucuronide conjugates and their hydrolysis, has been identified as one reason PBPK modeling has exceptional utility for UGT substrates [13].

PBPK modeling of Fg for UGTs has been limited, possibly due to limited clinical data elucidating Fg for UGT substrates, as well as challenges studying metabolism for these enzymes in vitro [13,14,15]. There are challenges with in vitro data, with microsomes in particular underestimating clearance [13]. Hepatocytes, both in short-term suspension (fresh and cryopreserved) and in a long-term co-cultured assay, may be useful in vitro tools for in vitro–in vivo extrapolation, IVIVE [16,17]. However, even hepatocytes may underestimate clearance for UGT substrates [18]. UGT in vitro assay conditions often impact results [14,19], and recent work has aimed at optimizing conditions (more details in Section 4). Even reaction phenotyping for UGTs can be challenging [19]. Intestinal microsomes have been demonstrated to be a potentially useful tool for predicting UGT intestinal metabolism [20], but this assay is challenging, particularly for more metabolically stable compounds. Challenges for UGT IVIVE also include organ-dependent specificity as well as clinical relevance for polymorphisms [21,22]. Nonetheless, recent work has demonstrated the potential utility of PBPK modeling to predict UGT-mediated intestinal metabolism through an IVIVE approach [16,23] and a focus on clinical absorption, distribution, metabolism and excretion (ADME) and PK data [24].

In this review, scientists from multiple companies participating in the GastroPlus^®^ User Group Steering Committee review examples of PBPK models for UGT substrates with a focus on Fg predictions. The incorporation of intestinal metabolism in PBPK models is described. The data available to parameterize the UGT levels in the GI tract are reviewed along with in vitro data useful for studying UGT metabolism to aid in model parameterization. Additionally, PBPK-DDI predictions for UGT1A1 substrates raltegravir and dolutegravir with UGT1A1 inhibitor atazanavir are included in these examples to illustrate the role of intestinal metabolism for DDIs. Although GastroPlus was used for all the examples, the approaches and information are generally applicable to PBPK modeling for predicting Fg for UGT substrates. Along with these industry examples, limitations of the approach are described. Other examples of UGT-mediated intestinal metabolism impacting exposures in the clinic are presented. Finally, the potential utility of an in silico tool for predicting substrate specificity for various UGTs is discussed in the context of its utility for application to PBPK modeling for prediction of first-pass loss from intestinal metabolism and bioavailability.

## 2. Incorporating Intestinal Metabolism in PBPK Models

The following section describes the GastroPlus PBPK model because it was used in the examples in Section 5. Other PBPK modeling tools, e.g., Simcyp^®^ [25] and PK-Sim^®^ [26], include similar physiological processes, although there are some differences in how different aspects of physiology are incorporated in the model.

UGTs are among the most important Phase II metabolic enzymes. They conjugate glucuronic acid with hydroxy groups, carboxylates, amines, heterocyclic nitrogens, and other nucleophilic centers in xenobiotics. Such polar groups generally reduce passive intestinal absorption. Often, drug candidates are designed to have high passive permeability by avoiding highly polar functional groups, and glucuronidation occurs at sites that are often introduced by phase I oxidation—hence the term “Phase II.” However, for some drugs, direct glucuronidation is the primary mechanism of elimination. PBPK modeling is a potentially useful tool for understanding the ADME of such compounds.

The advanced compartmental absorption and transit (ACAT) model describes the local solubility, dissolution, precipitation, absorption, metabolism, and active transport in each region of the intestinal tract for drugs administered orally. A compound must dissolve before it is available for absorption. Drug dissolution in individual intestinal compartments is calculated in ACAT by taking into account the amount and the composition (pH and bile salt concentration) of fluid in each intestinal compartment, the compartment-specific solubility and diffusion coefficient of a compound, and the formulation properties (e.g., size, shape, and density of the dissolving API particles, or the release rate from a modified release formulation). The dissolved compound can undergo precipitation and/or degradation inside the gut, which can lower the amount of compound available for absorption, and these processes can be parameterized based on in vitro experiments.

The absorption model includes passive transcellular and paracellular diffusion and carrier-mediated transport. The first-order absorption rate coefficients (units of s^−1^) in individual intestinal compartments are calculated from the product of effective permeability (units of cm/s) and absorption scale factors (units of cm^−1^), which account for changes in the drug’s ionization and absorptive surface area in the different parts of the small intestine and colon [27,28]. The carrier-mediated transport processes are described via transporter kinetics (Vmax, K_m_ values) and local gut transporter expressions. Intestinal transporters can decrease (efflux) or increase (influx) the amount of compound entering the enterocyte from its passive transcellular apical absorption. Similar processes exist on the basolateral membrane for passive or transporter-mediated movement into the portal vein.

Once a compound gets absorbed into intestinal enterocytes, it may be subject to gut metabolism. This first-pass process can lower the amount of drug entering the portal vein and then liver, impacting bioavailability.

The ACAT model describes intestinal first-pass metabolism due to UGT enzymes using Michaelis–Menten kinetics with K_m_ and Vmax values typically determined from in vitro experiments and converted to in vivo values. Then, built-in expression levels of specific UGT enzymes in different intestinal compartments are used in conjunction with the provided K_m_ and Vmax parameters to determine the extent of compound extraction in these gut regions and the potential for saturation with increasing dose levels. The region-specific intestinal expression levels of UGT enzymes in the ACAT model are defined as fractions of the total expression level of each enzyme in the entire liver if the enzyme is expressed in gut and liver, or as a relative expression in gut compartments if only the gut is involved.

The human ACAT and PBPK model has built-in expression levels of UGTs that are tissue-specific. For example, some UGTs that are expressed in the liver are not expressed in the gut, e.g., UGT1A7, UGT1A8, and UGT1A10 [29]. Additionally, some UGTs that are expressed in the liver and gut may not be significantly expressed in the kidneys (e.g., UGT1A1 [30]). Therefore, simulations with an appropriately parameterized model may clarify the organs involved with metabolizing a drug.

The interplay between processes taking place in the gut and their dependence on compound properties creates a complex picture of drug absorption. The standard output in GastroPlus includes the fraction of dose dissolved, Fa, FDp (i.e., fraction of dose passing into the portal vein, = Fa × Fg), F, area under the plasma concentration–time curve (AUC), maximum observed total plasma concentration (Cmax), and Cp-time profile, but many other simulation results, e.g., metabolite produced by a specific enzyme in a specific organ, can easily be incorporated. Given the potential complexity with additional mechanisms impacting systemic PK, PBPK modeling may be a useful tool for an integrated understanding of properties impacting bioavailability and PK in the hands of an experienced modeler.

## 3. UGT Expression in the GI Tract

The intestinal, hepatic, and renal UGT expression levels available in the current human ACAT model and human physiology in GastroPlus are based on a combination of protein and mRNA level information collected from four publications [9,10,31,32]. The metabolic enzyme abundance in the segments of the ACAT model is expressed relative to the total hepatic abundance in a typical adult subject based on relative intestine and liver weights and microsomal protein yields, as summarized by Soars et al. [33]. In the case that an enzyme is not present in the liver, the intestinal abundance is represented relative to the whole small intestine instead. A homogenous UGT enzyme abundance per intestinal surface area in the small intestines is assumed.

Experimental data on the variation of UGT protein abundance along the intestine are limited and partially inconsistent. Drozdzik and coworkers [34] found that measurements of UGT protein levels in tissue homogenates suggested a tendency towards higher UGT1A1, UGT1A3, and UGT2B7 concentration in mucosal tissue from jejunum than from duodenum or ileum. In contrast, Zhang and coworkers [35] reported relative UGT1A1, UGT1A3, UGT1A10, UGT2B7, and UGT2B17 protein abundance data suggesting a tendency towards highest UGT abundance in the duodenum and decreasing levels towards the more distal parts of the small intestine. However, this result was obtained when normalizing to the protein abundance of enterocyte marker proteins sucrase isomaltase and villin-1 in cryopreserved human intestinal mucosa samples, whereas without normalization to enterocyte markers, the same absolute UGT enzyme protein abundance data did not show clear differences in UGT abundance between the small intestine segments. Similarly, the results reported by Couto et al. [36] did not show statistically significant differences in UGT1A1, UGT1A3, UGT1A6, and UGT2B7 protein abundance between different segments of the small intestine. Consequently, the UGT abundance profiles in the human ACAT model, built in GastroPlus version 9.8, can be considered to be reasonably well supported by these experimental data.

When used in the context of PBPK models for the prediction of drug metabolism in tissues, both mRNA expression and protein abundance levels serve as surrogates for enzyme activity. However, for prediction of drug metabolism, protein abundance is generally preferred over mRNA expression [36] since it is more closely linked to enzyme activity. Post-translational regulation and the dynamic nature of protein expression in high turnover tissues such as the gut wall may mean that mRNA expression and protein abundance levels do not correlate well. Consequently, a significant body of liquid-chromatography–mass-spectrometry-based UGT protein abundance data in human tissues has emerged in the literature during recent years [8,11,34,35,36,37,38]. Based on the mean intestinal protein levels reported in the literature, as reviewed in [39,40], the distribution of UGTs in the intestine is UGT2B17, 52.2% > UGT1A1, 14.6% > UGT2B7, 9.2% > UGT1A10, 7% = UGT1A8 7%. However, there are considerable study-to-study differences in the reported quantitative UGT protein abundance in the gut wall. Differences in experimental practices on applying quantitative liquid chromatography–mass spectrometry proteomic techniques and consequent variability in reported abundances of the same proteins has been acknowledged [41] and efforts towards harmonized guidelines for LC–MS proteomic experimental practices and data analysis have been made [42]. Although part of the variability in reported absolute protein abundance data may be attributed to true interindividual variability between donors, the currently existing data on absolute UGT abundance in the human intestine are likely to be associated with considerable technical variability and quantitative uncertainty. Consequently, combining absolute protein abundance data from various studies may result in biased estimates of mean abundance and skew the estimation of interindividual variability.

In addition to abundance of enzyme protein in samples obtained via liquid chromatography–mass spectrometry (LC–MS) quantification, suitable scaling factors are required to estimate the total enzyme abundance in the whole tissue. The weight of the small intestine mucosal tissue (120 g) has been estimated to equate to approximately 8% of total liver tissue weight (1500 g) [43]. Correspondingly, the total amount of microsomal protein in the small intestine has been estimated to correspond to approximately 4 to 10% of total hepatic microsomal protein in the human liver [33,43]. The levels of UGT abundance in the small intestine relative to that in the liver were calculated assuming results on protein abundance and RNA levels in the liver and intestine are subject to a similar (albeit potentially unknown) level of quantitative bias when a similar methodology is used for samples from both tissues (Table 1). Comparison of the intestine-to-liver ratios based on several literature sources suggests that intestinal level estimates for UGT1A1 are reasonably consistent between several sources of experimental data, and the description in the ACAT model has a strong foundation. However, more limited and less consistent information is available for the other UGT enzymes. Potentially lower intestinal levels of UGT1A4, UGT1A6, UGT1A9, UGT2B7, and UGT2B17 than in the current ACAT model could be justified based on the existing protein abundance data on these enzymes. UGT expression profiles in the large intestine described in the current ACAT model are based on mRNA levels [10]. Data on UGT protein expression in the colon are limited. The majority of publications reporting LC–MS data on intestinal UGT protein levels have focused on the small intestines and those addressing UGT levels also in the colon [34] have reported levels below the limit of quantification. As more is learned about the expression and activity of the various UGTs in the GI tract, the parameters may be updated to match the state of the science.

## 4. In Vitro Methods to Measure UGT-Mediated Drug Metabolism

In vitro data on hepatic drug metabolism are generally required in drug development, whereas dedicated in vitro data on intestinal metabolism are likely to be generated on an ad hoc basis. Therefore, a reasonable approach to predict intestinal metabolism and to parameterize intestinal metabolism in PBPK models is to leverage in vitro methods established for prediction of hepatic metabolism and combine this information with reaction phenotyping data and information on the relative abundance of metabolic enzymes in the liver and intestine [2].

Several well-established in vitro methods are available for studying hepatic metabolism. Subcellular fractions such as microsomes may be used for studying metabolism by enzymes present and active in the given fraction, whereas primary human hepatocytes, either in suspension or as coculture models, represent holistic models for the metabolic machinery of the liver [44,45,46]. UGTs may be studied using microsomes fortified with uridine 5′-diphosphoglucuronic acid (UDPGA). However, clearance of UGT substrates in vitro using microsomes or other subcellular fractions has generally been associated with underprediction of hepatic clearance in vivo, and these underpredictions may be attributed to several mechanisms [13,18]. To overcome tendency towards underprediction, several details in microsomal incubation conditions have been explored and optimized, including preincubation of microsomes with pore forming agent alameticin to improve the access of UDPGA to the enzymes, supplementing incubations with albumin to bind UGT inhibiting free fatty acids, and optimization of incubation buffer constituents [19,47,48,49]. The activity of different UGT enzymes is not always similarly affected by variables of incubation conditions, and thus standardization of incubation conditions is needed and has been suggested [50]. However, the suggested experimental conditions may not be generally accepted, and thus uncertainty remains as to whether the contribution of individual UGT enzymes to microsomal clearance quantitatively resembles the relative contributions of these enzymes in vivo.

Due to potential limitations in the data generated with hepatic cellular fractions, intact primary human hepatocytes may be considered a gold standard for predicting hepatic clearance. Clearance measured in vitro with cryopreserved primary hepatocytes in suspension seems to be associated with a similar tendency towards clearance underprediction independent of whether the compounds are primarily eliminated via CYP or UGT enzymes [51]. This similar tendency suggests that the prediction of hepatic UGT-mediated clearance may not be associated with higher uncertainty than the prediction of CYP-mediated clearance. Cryopreserved primary hepatocytes in suspension are suitable for relatively short incubations and thus have limited utility for low-clearance compounds. Therefore, coculture models, such as Hµrel^®^ and HepatoPac^®^, including primary human hepatocytes cocultured with supporting cell types to allow longer-term incubations with consistent metabolic activity and hepatocyte viability throughout incubation, have been established. Evaluation of these coculture models have focused initially on compounds metabolized primarily via CYP enzymes [45,52,53], but promising quantitative prediction performance has also been demonstrated with compounds metabolized primarily by UGTs [16].

In addition to total metabolic clearance, utilization of data on hepatic metabolism for prediction of intestinal metabolism requires reliable reaction phenotyping data to specify the contribution of individual enzymes on hepatic metabolism and information on relative enzyme abundance in the liver and intestine. UGT protein levels in the intestine, relative to those in the liver, are discussed in Section 3. Use of enzyme-specific chemical inhibitors in incubations with an in vitro model for hepatic metabolism is accepted as one of the standard methods for cytochrome P450 reaction phenotyping, whereas a similar approach for UGT phenotyping is partially limited by a lack of generally accepted selective chemical inhibitors [19]. Another standard method for estimating contributions of individual UGTs on metabolism is to use recombinant UGT enzymes. The advantages of recombinant enzyme assays include certainty of the assay enzyme specificity and capability of addressing extrahepatic enzymes, such as UGT1A7, UGT1A8, and UGT1A10. However, using recombinant enzyme data to predict metabolism requires adequate IVIVE, taking into account the active enzyme content in vitro and in vivo, and also correction for potential differences in intrinsic enzyme activities between in vitro incubations and tissues in vivo. Intersystem extrapolation factor (ISEF) and relative activity factor (RAF) approaches have been utilized for this purpose with CYP enzymes [54,55,56] and a logical approach is to establish similar scaling factors for UGTs. Recent advances to establish RAF for UGTs have been made [57], but extrapolation of enzyme activity from recombinant enzymes to the in vivo situation is likely to be associated with higher uncertainty for UGTs and other phase II enzymes than for CYP enzymes. Furthermore, work to establish and evaluate RAFs for UGTs has been limited to hepatic UGTs; thus, IVIVE from recombinant enzyme data for extrahepatic UGTs requires further investigation.

The prediction of intestinal metabolism by scaling from hepatic clearance is bound to omit the contribution of enzymes present in the gut wall but not in the liver. Thus, the prediction of the intestinal metabolism of compounds eliminated via extrahepatic enzymes may benefit from in vitro methods specifically addressing intestinal metabolism. In vitro methods to study intestinal metabolism are often associated with high variability [58]; additionally, the relatively low metabolic activity in intestinal in vitro models may limit the utility of these methods to compounds with relatively fast metabolic turnover. Some promising examples of predicting intestinal UGT metabolism based on intestinal microsome data have been published [20]. However, use of intestinal microsomes for UGT metabolism may be expected to have similar limitations to the use of liver microsomes for the prediction of hepatic metabolism of UGT substrates. Consequently, recently published work on intestinal cryopreserved primary human enterocytes and cryopreserved human intestinal mucosal epithelium provides interesting and theoretically holistic in vitro models for intestinal metabolism, including both CYP and non CYP enzymes [35,59,60,61]. However, information on quantitative prediction performance of these novel intestinal metabolism in vitro models is currently still limited.

## 5. PBPK Modeling of UGT Intestinal Metabolism

The following case studies review PBPK models for UGT substrates with consideration of intestinal metabolism (Table 2). All the examples were done in GastroPlus.

### 5.1. Lorazepam (UGT2B7, UGT2B15), Oxazepam (UGT1A9, UGT2B15), Naloxone (UGT2B7), and Zidovudine (UGT2B7, CYP3A4)

Recently, Docci et al. [23] provided a detailed description of PBPK model construction for four UGT substrates: benzodiazepines lorazepam (UGT2B7, UGT2B15) and oxazepam (UGT1A9, UGT2B15), the antiretroviral zidovudine (UGT2B7, CYP3A4), and the opioid antagonist naloxone (UGT2B7). Docci followed a systematic model building approach, leveraging intrinsic clearance measurements made with HepatoPac^®^ [62], which is a micro-patterned coculture of primary human hepatocytes with mouse fibroblasts. Improved IVIVE performance for hepatic UGT substrates had been demonstrated in a companion paper from the same authors [16]. Docci et al. combined the intrinsic hepatic clearance estimates from Hepatopac with additional in vitro phenotyping data and published clinical mass balance studies to build PBPK models with the total systemic glucuronidation clearance apportioned to specific UGT isoforms expressed in liver and kidney. The estimation of relative contribution to systemic clearance of liver and kidney thus relied on the physiological model framework and the UGT isoform expression levels included as defaults in GastroPlus V9.7. After verification of systemic clearance simulations using clinical studies performed with intravenous dosing, the models were applied to simulate oral pharmacokinetics. Intestinal metabolism was estimated assuming an unbound fraction in the enterocyte of 100% and default intestinal UGT isoform regional expression ratios. These ratios scale the Vmax values for each UGT isoform in each gut compartment relative to the amount of that isoform in a typical human liver.

Results for the default simulations of oral pharmacokinetics were interesting, as a consistent underprediction of bioavailability was apparent for three of the four substrates, namely lorazepam, oxazepam, and zidovudine. For lorazepam, 81% was predicted with 93% observed; for oxazepam, 79% was predicted compared to 92% observed; and for zidovudine, 40% was predicted compared to 63% observed. Parameter sensitivity analyses revealed that variations in the enzyme activity in the gut (Gut Vmax) caused a relatively high change in the simulated Fg for these three models, while other tested parameters (permeability, solubility, fu in enterocytes) were less sensitive (<10% change). Hence, when the models were adjusted by applying a consistent scaling factor of 0.25-fold to the Gut Vmax values for the involved UGT isoforms (UGT2B15, UGT2B7, and UGT1A9), the simulated intestinal metabolism was reduced, and bioavailability estimates and oral profiles agreed better with the observed data. For naloxone, a similar approach was not fruitful since, in contrast to the other three drugs, naloxone has low bioavailability due to a high hepatic extraction ratio, making it more sensitive to hepatic metabolism changes than to intestinal metabolism. 

### 5.2. Cabotegravir (UGT1A1, UGT1A9)

Cabotegravir is a potent integrase inhibitor, particularly useful against many subtypes of human immunodeficiency virus 1 (HIV-1) and is an analogue of dolutegravir. Cabotegravir is a moderately lipophilic low solubility compound in its neutral form with <10 ug/mL solubility [64]. However, with a pKa of 4.52 (acid), it is almost completely ionized in the small intestine, and the solubility at pH 6.5 is 0.83 mg/mL based on a combination of in silico and in vitro measurements. Cabotegravir has essentially no dissolution or precipitation limitations at its clinical dose of 30 mg when administered as a solution, as was done in the studies used for model development. Additionally, cabotegravir has a predicted human effective jejunal permeability of 2.54 × 10^−4^ cm/s, which puts it the high permeability category. Because there are no dissolution (due to solution formulation), precipitation, or absorption limitations, cabotegravir is an excellent candidate to analyze UGT metabolism in the absence of any external complicating factors. The primary route of metabolism of cabotegravir is via glucuronidation by UGT1A1 and UGT1A9 [64].

PBPK simulations were conducted in GastroPlus^®^ 9.6 (Simulations Plus, Inc, Lancaster, CA) and ADMET Predictor^®^ 8.1 (Simulations Plus, Inc., Lancaster, CA, USA) was used to estimate most physicochemical inputs. Additional inputs were either experimentally determined values or substituted values from analog compound dolutegravir, which is only one carbon atom different from cabotegravir. Table 2 summarizes the key inputs for the cabotegravir model. The Lukacova model was used to calculate all tissue partition coefficients and passive kidney filtration was employed based on fraction unbound in plasma (fu,p) and glomerular filtration rate (GFR). The fu,p and blood-to-plasma ratio (Rbp) were estimated from whole blood and plasma radioactivity experimental data in the literature [64]. UGT1A1 and UGT1A9 gut and liver metabolism was described using in vitro values [64]. The in vitro K_m_/Vmax values were converted to in vivo values for gut and liver using the built-in IVIVE conversion tool that utilizes all default expression levels. Human liver microsomes (HLMs) with recombinant UGT1A1 and 1A9 enzymes at a concentration of 0.5 mg/mL were used to estimate drug metabolism using the Halifax method for in vitro binding. K_m_ and Vmax values for in vitro metabolism of UGT1A1 and 1A9 were 148 and 90 uM and 660 and 200 pmol/min/mg protein, respectively [64]. Default gut and liver UGT expression levels were used as described in Section 3.

**Figure 1:**
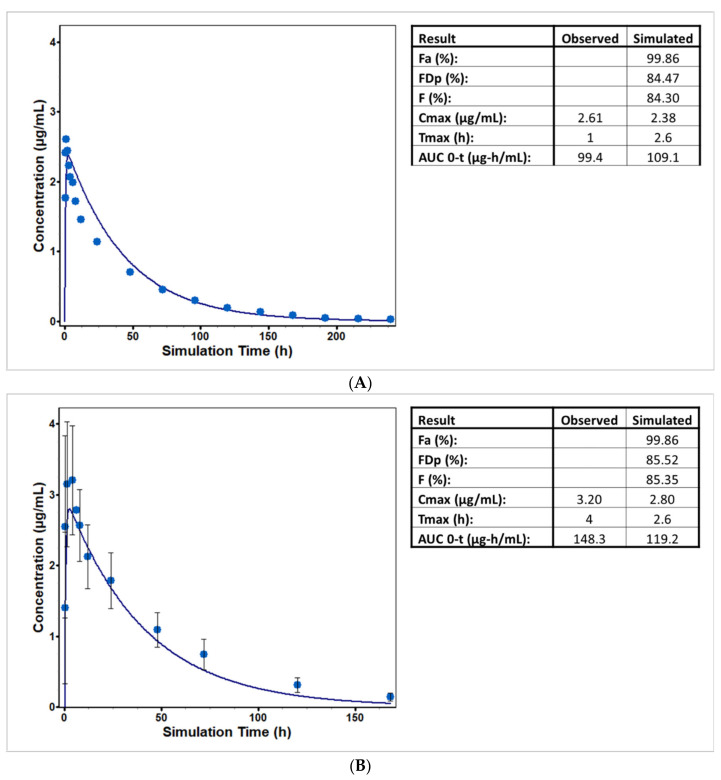
PBPK Model prediction of (**A**) 28.2 mg and (**B**) 30 mg oral solution of cabotegravir. Dark blue curves and light blue points represent predicted versus observed plasma concentration. The tables list: fraction of dose absorbed (Fa), fraction of dose reaching the portal vein (FDp), bioavailability (F), maximum observed total plasma concentration (Cmax), time at which the Cmax was observed (Tmax), and the area under the plasma concentration–time curve from time zero to t (AUC_0–t_).

A combinatorial approach utilizing in vitro and in silico data was then utilized to predict the in vivo concentration versus time profile of two oral solution formulations, as shown in Figure 1. Study 1 is a 28.2 mg oral solution administered to six male subjects with an average age of 41.2 yr and weight of 88.8 kg [64]. The observed versus predicted Cmax (2.6 and 2.38 ug/mL) and AUC (0–240 h) (99.4 and 109.1 ug·h/mL) are within 10% error, a reasonable result when considering the IVIVE extrapolation of metabolism. Study 2 is a 30 mg oral solution dosed in both male and female subjects, and an average weight of 79.63 kg was used to generate the PBPK physiology [65]. The observed and predicted Cmax (3.2 and 2.8 ug/mL) and AUC (0–168 h) (119.2 and 148.3 ug·h/mL) are within 20% error in this case. In both cases, almost all first-pass extractions are attributable to gut first-pass extractions at 15.4 and 14.3%. Hepatic first pass extraction is very low and approximately 0.18% in both cases. This modeling result is from the high unbound enterocyte concentrations relative to the low unbound liver concentrations due to the low fraction unbound in plasma. 

Limitations of the current modeling approach include lack of an intravenous, IV, PK study in humans, measured permeability data, and experimentally determined LogP or LogD vs. pH profile. Of these limitations, the IV data is key. While the in vitro data for metabolism and the PBPK approach, in this case, provided accurate Cp-time predictions, the overall first-pass effect cannot be assessed with full certainty without PK data from IV administration.

### 5.3. Dolutegravir (UGT1A1, CYP3A4)

Dolutegravir, a HIV-1 integrase inhibitor, is a Biopharmaceutics Drug Disposition Classification System class two drug. It is a substrate for the P-glycoprotein (P-gp) and breast cancer resistance protein (BCRP) transporters but exhibits rapid oral absorption and dose-proportional kinetics. These properties suggest that dolutegravir absorption is mainly governed by its high intrinsic intestinal permeability with no major impact of P-gp and BCRP. Dolutegravir metabolism was determined in vitro to be mainly metabolized by UGT1A1 (fraction of metabolism mediated by UGT1A1, f_m,UGT1A1_ = 0.51) and cytochrome P450 3A4 (f_m,CYP3A4_ = 0.21). UGT1A3 and UGT1A9 are only minor pathways (f_m,UGT1A3_ = 0.028 and f_m,UGT1A9_ = 0.055) [63]. Less than 1% of unchanged drug is excreted urine [66].

The dolutegravir PBPK model was developed and validated in GastroPlus™ v9.0. In the model, the dolutegravir metabolic clearance was assumed to be mediated by the UGT1A1 and CYP3A4 enzymes only, and since P-gp and BCRP impact was expected to be negligible, they were not included in the model. 

**Figure 2:**
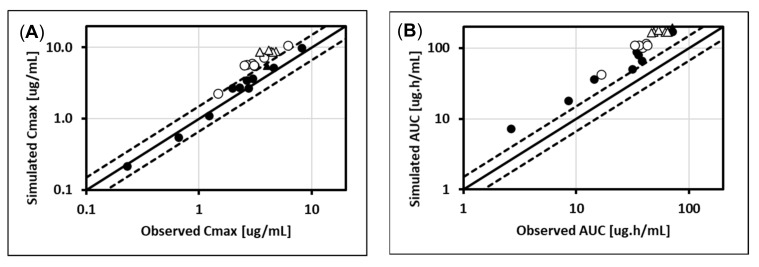

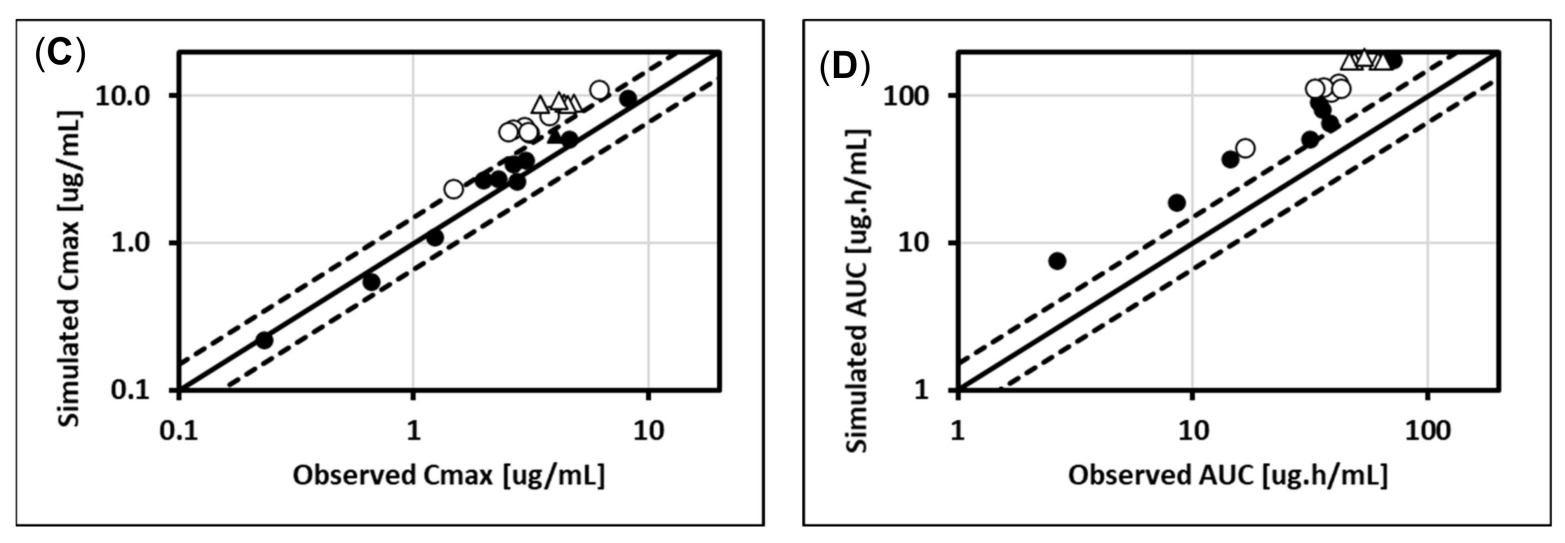
Comparison of simulated to observed Cmax and AUC (AUC_0–t_ or AUC_tau_) from different published studies [67,68,70,71,72,73,74,75,76,77,78] with predictions based on in vitro parameters for dolutegravir interaction with UGT1A1 measured in recombinant UGT1A1 (**A**,**B**) and in HLM (**C**,**D**). Circles represent fasted studies, triangles represent studies in which dolutegravir was administered with a moderate-fat meal, closed symbols represent single dose, and open symbols represent steady-state data. In each plot, the solid line represents the identity line, and the dashed lines show margins for 1.5-fold errors. AUC = the area under the plasma concentration–time curve, AUC_0–t_ = AUC from time zero to t, AUC_tau_ = AUC during the dosing interval, Cmax = maximum observed total plasma concentration, and HLM = human liver microsomes.

Dolutegravir K_m_ and Vmax values for UGT1A1 were available from in vitro measurements in HLMs and recombinant human UGT1A1 (rUGT1A1) [63]. The K_m_ and Vmax values measured in HLMs were approximately six- to seven-fold higher than values measured in rCYP, but very similar intrinsic clearances were measured in the two systems (2.7 µL/min/mg and 3.2 µL/min/mg measured in HLMs and rUGT1A1, respectively). The in vitro K_m_ and Vmax were used in the initial model parameterization along with built-in expression levels of both enzymes in gut, liver, and kidney. The data from both in vitro systems underpredicted the in vivo clearance (Figure 2). The single dose Cmax values were predicted with reasonable accuracy (average prediction error 13%). However, the average prediction errors for steady-state Cmax as well as single dose and steady-state AUCs were more significant (two- to three-fold overprediction). Therefore, the model was further refined by fitting some of the parameters against clinical data after 25, 50, and 100 mg single dose administration in fasted subjects [67] and a crossover study with 50 mg single dose administration in fasted and fed (moderate-fat meal) subjects [68] and by utilizing information from mass balance and DDI studies [69]. 

**Figure 3:**
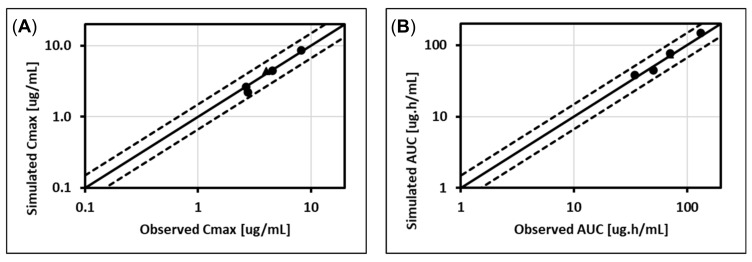

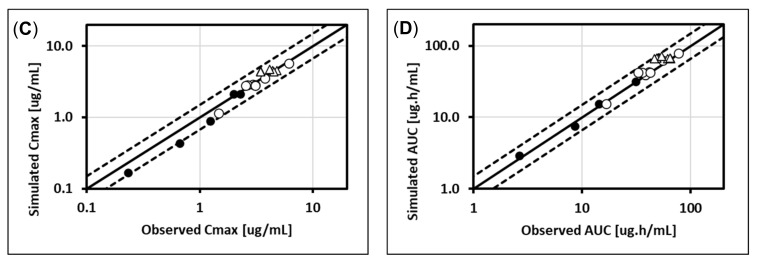
Comparison of observed Cmax and AUC (AUC_0–t_ or AUC_tau_) from different published studies [67,68,70,71,72,73,74,75,76,77,78] with final model simulations for studies used for model development (**A**,**B**) and model validation (**C**,**D**). Circles represent fasted studies, triangles represent studies where dolutegravir was administered with a moderate-fat meal, closed symbols represent single dose data, and open symbols represent steady-state data. In each plot, the solid line represents the identity line, and the dashed lines show margins for 1.5-fold errors. AUC = the area under the plasma concentration–time curve, AUC_0–t_ = AUC from time zero to t, AUC_tau_ = AUC during the dosing interval, and Cmax = maximum observed total plasma concentration.

Subsequently, the model was validated by predicting dolutegravir PK after 2–50 mg single dose administrations in fasted subjects [67,70,71,74] and 10–50 mg multiple-dose administrations in fasted [67,70,71,73,75] and fed subjects [72,75,76,77,78]. The average prediction error for Cmax and AUC_inf_ was 1 and 5%, respectively, for studies used for model development. The average prediction error for Cmax and AUC (AUC(0-t) for single dose and AUCtau for multi-dose administrations) was 4 and 11%, respectively, for studies used for model validation. The final model (Figure 3) used an in vitro K_m_ measured in rUGT1A1 and Vmax values fitted against clinical data. For a 50 mg dose of dolutegravir, nearly 18% of the dose was metabolized by the gut, with UGT1A1 contributing approximately 80% to the intestinal metabolism and CYP3A4 contributing the remaining 20%. The contributions of intestinal metabolism came from the clearance calibration against in vivo PK profiles of dolutegravir and the default intestinal and liver expression levels of both enzymes. 

After the dolutegravir PBPK model was refined and validated, it was used to predict the DDI with atazanavir, a UGT1A1 reversible inhibitor and a competitive inhibitor as well as a mechanism-based inhibitor of CYP3A4. The DDI with atazanavir [69] was predicted accurately, with predicted effect (ratio of dolutegravir Cmax and AUC when administered with atazanavir and alone) within 16% of the observed ratios of 1.54 for Cmax and 1.92 for AUC. This accurate prediction confirms the appropriate calibration of UGT1A1 intestinal contribution in the dolutegravir clearance.

The PBPK atazanavir model was developed and validated using data from seven clinical studies in healthy volunteers, as described by Reddy et al. [24]. The total inhibition constant, Ki, for UGT1A1 was 1.9 µM [79] and the unbound fraction in microsomes measured in vitro of 0.502 was incorporated. For CYP3A4, the Ki was estimated using the atazanavir K_m_ of 0.362 µM [80], adjusted for microsomal binding; the mechanism-based inhibition of CYP3A4 by atazanavir was included using K_I_ = 0.641 μM; and the rate of enzyme inactivation, k_inact_ = 0.114 min^−1^, was derived from the concentration-dependent inhibition of CYP3A4 in human liver microsomes, with and without preincubation with atazanavir [81]. Atazanavir is a hepatic uptake transporter substrate [80], and the PBPK modeling for atazanavir did not explicitly incorporate the role of transporters for atazanavir. Importantly, Nicolai et al. [82] studied the interplay of transporters and metabolism for atazanavir in rats in vitro, and found that involvement of active uptake transport did not cause high intracellular levels in this case. For atazanavir, they found that the ratio of unbound intracellular to extracellular concentration in hepatocytes was approximately 0.3, and likewise, the unbound Michaelis–Menten constant, K_m,u_, was >three-fold higher in hepatocytes than microsomes. For this reason, Ki values for atazanavir observed in vitro can depend on the system used. But the ability to predict the DDI between atazanavir and dolutegravir, and raltegravir (see Section 5.4), improves confidence in the atazanavir model.

The dolutegravir final model provided an excellent description of its PK, but the clinical PK data and ADME data were necessary to properly calibrate the elimination due to uncertainties in the reported in vitro values as well as the contribution of CYP3A4 to dolutegravir metabolism, for which no in vitro data were available. Interestingly, with the in vivo K_m_ value for UGT1A1 fixed at the reported in vitro K_m_ value, measured in recombinant UGT, the fitted Vmax that correctly described the in vivo DGT clearance was close (~30% lower) to the in vitro Vmax measured in HLM. The difference in K_m_ values measured in the two in vitro systems could potentially be due to the higher nonspecific binding of dolutegravir in HLM, and the difference in Vmax values might suggest the different activity of rUGT1A1 from that of UGT1A1 in the HLM system (which might more closely resemble the in vivo activity).

### 5.4. Raltegravir (UGT1A1)

Raltegravir is an HIV-1 integrase strand transfer inhibitor also used in combination with other antiretroviral agents. The raltegravir PBPK model, described by Reddy et al. [24], was built using GastroPlus version 9.0. It was modified from a preliminary model provided by Simulations Plus.

The raltegravir model incorporated in vitro and in vivo data, with a focus on matching available clinical PK and ADME data. The model incorporated clearance of raltegravir through UGT1A1 metabolism (mainly through hepatic metabolism, since UGT1A1 expression in the kidney is relatively low [30]) and renal elimination (~9% estimated as fu × GFR) based on human ADME data described in the Isentress^®^ label [83]. UGT1A1-mediated intestinal metabolism was incorporated in the model through specifying UGT1A1 expression in the gut (default parameters, see Section 3). The UGT1A1 K_m_ value for raltegravir was based on an in vitro measurement [84]. However, the Vmax values for the liver and gut were optimized to match clinical data. Raltegravir PK data exhibited significant differences (up to ~three-fold) in exposures between studies. It was assumed that the differences in exposure were likely due to differences in absorption, i.e., related to different formulations; therefore, for studies with significantly lower exposures (~three-fold), properties leading to poor absorption (i.e., higher particle radius and decreased solubility) were assumed.

The raltegravir model was developed to determine whether the DDI with UGT1A1 inhibitor atazanavir could be predicted. Details of the atazanavir model are described in [24], but a brief description is provided in Section 5.3. In general, the predicted increase in raltegravir exposures observed with atazanavir coadministration was reasonably accurate (Table 3), although the impact of the DDI was somewhat overestimated. Simulations indicate that the main reason atazanavir increases raltegravir exposures is the inhibition of raltegravir intestinal metabolism. For example, for the Zhu et al. [85] study, simulations indicate that Fg increased from 51–52% without, to 87% with atazanavir coadministration.

## 6. Discussion

### 6.1. Prediction of Intestinal Metabolism Mediated by UGTs with PBPK Modeling

The importance of intestinal metabolism to oral bioavailability is well studied but mainly for CYP substrates. For CYP3A4 substrates, many factors contributed to the success of developing PBPK modeling as a tool for predicting Fg. Yang et al. [4] developed a modeling approach to predict intestinal metabolism for CYP3A4 substrates using clinical data from the literature. They noted three types of studies that could be used to study the impact of CYP3A4 on oral bioavailability: (1) anhepatic patients, (2) comparison of exposure from IV and oral PK, and (3) comparison of PK with and without an inhibitor (e.g., studies with grapefruit juice co-administration, which at the right dose is thought to inhibit mainly intestinal CYP3A4) [4]. In vivo intestinal metabolism of midazolam (a CYP3A4 substrate) was studied following IV and intraduodenal administration during the anhepatic phase of liver transplantation [88]. However, to our knowledge, data for any UGT substrates in anhepatic patients do not exist. The grapefruit juice study also does not apply since it does not inhibit UGTs in the same way as for CYP3A4. Moreover, there are limited PK data for administration of UGT substrates by both IV and oral (PO) routes that can be compared for the determination of Fg (see examples in Section 6.2). Here, we propose a similar approach, which has been used to great effect for CYP3A4 substrates, but apply it to UGTs.

UGT substrates are often thought of as having low clearance. While this is often the case, UGT-mediated intestinal metabolism can significantly reduce exposure. The prediction of intestinal metabolism based on nonclinical data is critical for selecting a compound likely to achieve acceptable exposure in the clinic. Additionally, predicting intestinal metabolism is important for assessing a compound’s DDI liabilities [3]. Here, we described the use of PBPK models to predict intestinal metabolism, including underlying data such as expression levels in the intestines, and reviewed examples utilizing PBPK modeling to understand the extent of intestinal metabolism for drugs that undergo UGT metabolism.

### 6.2. Clinical Relevance of UGT Intestinal Metabolism

Clinical IV and PO PK data from the literature have been reported for UGT substrates, and were used to calculate Fg (with values either reported or determined using standard equations described by Kharasch et al. [89]), with results provided in Table 4. Mizuma et al. [12] described a greater impact of intestinal glucuronidation than hepatic glucuronidation on the bioavailability of raloxifene, a UGT1A1, 1A8, 1A9, and 1A10 substrate. Based on the reported human data of raloxifene, this study estimated an absolute F of 0.02 with an Fa of 0.63, Fg of 0.054, and Fh of 0.593 [12]. Estimated Fg values for other UGT substrates have also been reported in the literature [20,90] and are listed in Table 4. This analysis provides another way of estimating intestinal metabolism for UGT substrates but provides limited insight in compound properties leading to first-pass metabolism. 

Interestingly, for substrates including raloxifene, troglitazone, and diclofenac, Fg values are lower than Fh values, indicating greater first-pass loss from intestinal metabolism than from hepatic metabolism. An in vitro study identified UGT1A1 and 1A10 [91] as the enzymes responsible for the glucuronidation of troglitazone. UGT1A10 exhibited high catalytic activity and is expressed only in extrahepatic tissues (e.g., intestine and colon), which explains the low calculated Fg value for this compound. The major enzyme responsible for glucuronidation of diclofenac was UGT2B7, followed by 1A9 and 1A6 = 2B15 [92]. The subfamilies of UGT1A and UGT2B are expressed in both liver and intestine (see Section 3). The relatively high expression of UGT2B7 in the intestines seemingly resulted in a lower Fg value than Fh value for diclofenac [90]. In the case of acyl-glucuronide formation (e.g., gemfibrozil) enterohepatic circulation has been demonstrated due to the gut hydrolysis of the labile acyl-glucuronide, which may have resulted in Fa > 1.0. For example, the Fg = 1.09 for gemfibrozil in Table 4 is likely a reflection of an increase in Fa > 1.0. The estimation of Fg from clinical IV and PO PK data for these examples illustrates the range of potential impact of UGT-mediated intestinal metabolism on oral bioavailability.

This analysis also demonstrates that care is needed in interpretation of the Fg calculated using this method. For example, in the case of canagliflozin, the calculated Fg of 0.75 suggests that canagliflozin undergoes intestinal metabolism. However, canagliflozin is primarily metabolized by UGT1A9 and UGT2B4, and these isoforms are not expressed in the intestine [8]. Several assumptions in these calculations can lead to the over-estimation of intestinal metabolism, as has been discussed before [93]. In the case of canagliflozin, the discrepancy could be due to the assumed Fa of 1, which may well be an over-estimation given that canagliflozin is a BCS class IV molecule [94]. 

A clinical DDI study with a UGT inducer such as rifampin may not, by itself, be able to elucidate the contribution of intestinal metabolism for UGT substrates. For example, in DDI studies with rifampin, AUC for canagliflozin and raltegravir were reduced to a similar extent: 51% [95] and 40% [96], respectively. However, there are differences in the calculated Fg of 0.75 for canagliflozin (Table 4) vs. ~0.5 for raltegravir (Table 2). This example further highlights the challenges in elucidating the contribution of intestinal metabolism on a drug’s disposition. As such, a combination of PBPK modeling, use of mechanistic in vitro studies, and carefully designed clinical DDI studies may be used to better understand the underlying processes for intestinal first-pass effects.

Intestinal metabolism can be important from a DDI perspective. The raltegravir example (Section 5.4) highlights the role of intestinal metabolism in the DDI with the UGT1A1 inhibitor atazanavir. However, DDIs with rifampin, even when intestinal metabolism plays a role, do not seem to be as significant for UGT substrates as for CYP3A substrates. PBPK modeling is a potentially useful tool for assessing the DDI risk for UGT substrates, although limited probe substrates and well-characterized perpetrators are available for model qualification.

### 6.3. Limitations of the PBPK Approach for Predicting Fg for UGT Substrates

Data for UGT1A1 expression in the GI tract are reasonably consistent across studies, and therefore PBPK modeling for UGT1A1 intestinal metabolism has a stronger foundation than for other UGTs (e.g., UGT1A4, UGT1A6, UGT1A9, UGT2B7, and UGT2B17), for which existing expression data are less consistent (Table 1). In the modeling work of Docci et al. [23], initial predicted oral clearance based on IVIVE was within two-fold for lorazepam (substrate of UGT2B7 and UGT2B15), oxazepam (substrate of UGT1A9 and UGT2B15), and zidovudine (substrate of UGT2B7 and CYP3A4), but bioavailability was underestimated until Vmax values were scaled with an empirical factor of 0.25 to reduce the predicted first-pass metabolism in the gut. As additional intestinal expression data and modeling work becomes available, PBPK modeling for all the UGTs expressed in the GI tract will be strengthened by capitalizing on this systems biology approach.

Parameterizing saturable metabolism, which can be important for intestinal metabolism, particularly for high-dose compounds with high concentrations in the GI tract, can be a challenge for PBPK modeling of intestinal metabolism. Heikkinen et al. [6] evaluated the accuracy and precision of GastroPlus Fg predictions for a set of 20 CYP3A substrates using in vitro and in silico inputs. Overall, good Fg prediction accuracy was found (no significant bias and 95% of predictions within two-fold from in vivo estimated Fg), but the precision was limited, especially for high extraction compounds. Given the challenges of studying the metabolism of UGT substrates in vitro (see Section 4 and the dolutegravir example in Section 5.3), confidence in a Km estimate may be limited. Parameter sensitivity analysis for Km and for parameters impacting local drug concentrations in the GI tract (e.g., solubility, dissolution) may be important for understanding the impact of uncertainty on the Fg estimate. Therefore, in the early stages of drug development, a bottom-up PBPK approach to identify potential uncertainties and their impact on predictions would be recommended [2], but once clinical data are available the PBPK model may be refined via top-down fitting of nonlinearity (i.e., adjusting Km based on fitting to dose-dependent PK).

The modeling examples presented here are all relatively simple, for drugs that form primary glucuronide conjugates. The models did not include enterohepatic recirculation or transporter-mediated mass transfer, which may be needed for glucuronide conjugate metabolites to be included in the modeling. However, these models provide a foundation for others to build on with more complex examples.

Of course, applying PBPK modeling requires a solid understanding of the PK properties of a compound. To apply PBPK modeling prospectively before clinical data are available results in greater uncertainty. However, even when clinical data are available, the lack of specific types of data (e.g., mechanistic in vitro ADME data, human IV PK data, or human ADME study data may not be available) may lead to uncertainty [2,101]. Nonetheless, the examples presented in this paper show PBPK model utility for understanding intestinal metabolism for UGT substrates using an IVIVE approach as well as based mainly on clinical data.

### 6.4. In Silico Example: Predicted Substrate Specificity for UGT Enzymes

Knowing that a compound is subject to metabolism by Phase I, Phase II, or hydrolytic cleavage is key to understanding its bioavailability and, if it is a prodrug, its bioactivation. Prediction of which metabolites are most likely produced by these metabolic mechanisms is also critical to understanding the toxicology, efficacy, and pharmacokinetic properties of drug candidates. All the UGT substrates included in the examples in this publication have aliphatic or aromatic hydroxyl groups and do not require phase I metabolism prior to conjugation.

The Metabolism Module of ADMET Predictor^®^ (Simulations Plus, Inc., Lancaster, CA, USA) provides substrate classification models for nine human UGT isoforms. Much more data are available for CYP enzymes, and correspondingly, more detailed models are available for CYPs, including models for estimating kinetic parameters as well as ones for substrate and inhibitor classification. Substrate classification models are provided for human UGTs 1A1, 1A3, 1A4, 1A6, 1A8, 1A9, 1A10, 2B7, and 2B15. These models are classification artificial neural network ensemble models based on substrate specificity data from about 270 publicly available literature sources (e.g., [102,103]). The number of compounds used to train these nine models ranged from 196 to 319. Unfortunately, the combination of complex kinetics and scarcity of suitable data for UGT enzymes precludes the ability to provide kinetic models or estimate clearance for UGT enzymes. However, in silico models included with ADMET Predictor can generate a schematic map of metabolites most likely to be generated for each UGT for compounds that are predicted to be substrates of a given UGT.

For all drugs described in this manuscript and two more with well-characterized metabolism (canagliflozin and tapentadol), Table 5 provides the in silico predictions of substrate specificities for nine UGT enzymes (UGT1A1, UGT1A3, UGT1A4, UGT1A6, UGT1A8, UGT1A9, UGT1A10, UGT2B7, and UGT2B15). For each enzyme, the table includes “Yes/No” results regarding the predicted substrate specificity and includes “(% confidence)” that the classification model is accurate. The results are outlined with a red colored square for false positives, a green background for true positives, and a red “Yes/No” for molecules that were out of scope for that prediction. For example, cabotegravir was accurately predicted to be a substrate of UGT1A1 and UGT1A9 but also had a false positive substrate prediction for UGTs 1A8, 1A10, 2B7, and 2B15. It should be noted that the classification accuracy was very low at 56 and 52% for 1A10 and 2B15, respectively, and so the predictions may be less reliable. Only two molecules out of nine (canagliflozin and oxazepam) did not have an accurate prediction of substrate specificity. Only five out of 43 negative predictions were false negatives (i.e., predicted to not be substrates for enzymes with experimental proof of substrate specificity), but 38 out of 43 negative predictions were true negatives.

Overall, it can be concluded that the purely in silico predictions for UGT substrate specificity are quite accurate and would be useful in planning a limited number of additional experiments for confirmation. The results are encouraging and show promise for pure in silico predictions but will need to be confirmed with a larger set of compounds. Combining this in silico assessment with understanding of metabolite stability and knowledge of UGT expression in the GI tract may allow an initial assessment, qualitative and perhaps even quantitative through PBPK modeling, of the likelihood of decreased exposures from first-pass extraction due to intestinal metabolism.

## 7. Conclusions

PBPK modeling has become an important tool for estimating intestinal metabolism for CYP3A substrates, and here, we show that this valuable method can be used for UGT substrates. Protein and mRNA level data are available to estimate intestinal, hepatic, and renal UGT expression levels. Estimates for UGT1A1 expression in the intestines are reasonably consistent across several sources of experimental data, but for the other UGT enzymes, data are more limited and less consistent. As more is learned about the expression of UGTs, the parameters will be refined to leverage this systems biology approach. Although there are challenges in studying metabolism in vitro for UGT substrates, advances, such as coculture models including Hµrel^®^ and HepatoPac^®^ for low clearance compounds and better understanding of the best conditions for in vitro studies of UGT substrate metabolism, may provide opportunities for improved IVIVE. PBPK modeling has been shown to be a useful tool for modeling the intestinal metabolism of UGT substrates to understand compound properties contributing to first-pass losses, but additional examples with IVIVE would build confidence in prospective predictions. PBPK modeling shows promise as an increasingly useful tool for predicting first-pass loss from intestinal metabolism for UGT substrates.

## Figures and Tables

**Table 1:** Intestinal and hepatic UGT protein abundance and mRNA expression ^1^.

	Small Intestine (pmol/mg prot)	Liver (pmol/mg prot)	Small Intestine/Liver Ratio ^2^	Small Intestine (pmol/mg prot)	Liver (pmol/mg prot)	Small Intestine/Liver Ratio	Small Intestine/Liver Ratio	Small Intestine (pmol/mg prot)	Liver (pmol/mg prot)	Small Intestine/Liver Ratio	Small Intestine (Copy Number Normalized to GAPDH)	Liver (Copy Number Normalized to GAPDH)	Small Intestine/Liver Ratio	Small Intestine/Liver Ratio Assumed in ACAT Model Built in GP 9.8
UGT1A1	7.2	18.3	0.0157	39.6	124	0.0128	0.0280	0.33	0.87	0.0152	582	1430	0.0326	0.0196
UGT1A3	<0.5	9.9		1.93	20.6	0.0037	0.00284	0.04	0.3	0.0053	24	131	0.0149	NA
UGT1A4	5.3	4.6	0.0461	1.6	84	0.0008		<0.99	3.95		BLQ	618		0.0462
UGT1A5											72	9	0.667	0.401
UGT1A6	2.3	5.2	0.0177	<2	22.6			<0.22	1.02		95	468	0.0162	0.0188
UGT1A7 ^3^	8.4	BLQ		<2	<1						26	5	0.4150	4.52 ^3^
UGT1A8 ^3^	6.1	BLQ		<2	<1						70	BLQ		NA ^3^
UGT1A9	6.6	26.7	0.0099	<2	61.2			<0.03	1.43		38	1210	0.0025	0.0156
UGT1A10 ^3^	4.7	BLQ		17.9	<1			4.35	<1.91		968	BLQ		NA ^3^
UGT2B7				15.7	200	0.0031	0.00589	0.77	6.01	0.0051	1930	4220	0.0366	0.0268
UGT2B15				<2	99.7			<0.72	3.76		738	18,500	0.00319	0.00496
UGT2B17				112	54.3	0.0825		1.91	0.21	0.364	2680	197	1.09	0.655
Source	Harbourt et al. [9] ^4^			Sato et al. [11] ^4^			Drozdzik et al. [34] ^5^	Basit et al. [8] ^6^			Ohno and Shizuo [10] ^4^			
Sample type	Microsome fraction			Microsome fraction			Tissue homogenate	S9 fraction			Total RNA			

^1^ Results below the limit of quantification or limit of detection = BLQ. Uridine 5′-diphospho-glucuronosyltransferase = UGT. Empty cells indicate no data. ^2^ The small intestine-to-liver ratio is the ratio of the estimated total small intestine abundance to the total hepatic abundance using the data from the same study for both tissues. These are calculated assuming total microsomal (or S9) protein abundance in the small intestine is 4% of total microsomal (or S9) protein abundance in the liver. Total RNA samples are assumed to represent the UGT levels in intestinal mucosa; intestine to liver ratio is calculated assuming intestinal mucosa mass is 8% of liver mass. ^3^ UGT1A7, UGT1A8, and UGT1A10 are considered to be extrahepatic enzymes [29]. However, low hepatic UGT1A7 expression has been reported [10] and has been used for parameterizing the current GastroPlus physiology model. Otherwise, the intestinal abundance of extrahepatic enzymes is represented relative to the whole small intestine instead of relative to liver in the ACAT model. ^4^ Harbourt et al. [9], Sato et al. [11] and Ohno and Shizuo [10] report UGT levels in the human small intestine without specifying the part of small intestine used for sample preparation. ^5^ Drozdzik et al. [34] report UGT abundance in several segments of intestine and include estimates of total small intestine and liver UGT protein abundance. ^6^ Basit et al. [8] report UGT abundance in samples prepared from the first half of the small intestine.

**Table 2:** Summary of example UGT substrate PBPK models including key compound properties ^1^.

Parameter	Cabotegravir	Dolutegravir	Lorazepam	Oxazepam	Naloxone	Raltegravir	Zidovudine
Drug class	Antiretroviral	Antiretroviral	Benzodiazepine	Benzodiazepine	Opioid antagonist	Antiretroviral	Antiretroviral
Model reference	Internal Sim+	[24]	[23]	[23]	[23]	[24]	[23]
MW, g/mol	405.4	419.4	321	287	327	445.2	267
pKa	4.52 (acid)	4.58 (Acid), 8.2 (Acid)	1.3 (base) 11.5 (acid)	2.57 (base) 11.31 (acid)	7.9 (base)	5.5 (acid), 1.53 (base)	9.1 (acid)
LogP	2.16 ^2^	2.16	2.4	2.4	1.92	0.58	0.06
Solubility, mg/mL (reference pH)	<0.01 free acid	2.1 × 10^−4^ at pH = 1.2	0.0485 at pH = 7	0.057 at pH = 7	0.66 at pH = 8.45	0.014 (1.2) 0.020 (4.5) 0.1 (6.8)	25.4 at pH = 7
Permeability (cm/s × 10^4^)	2.54	2.37	3.3	3.6	4	1.79	1.9
fu,p	0.01	0.009	0.11	0.6	0.56	0.17	0.74
Rbp	0.58	0.55	0.75	1.1	1.1	0.60	0.86
Major CL mechanism	Metabolism (~79%), renal (~21%)	Metabolism	Metabolism	Metabolism	Metabolism	Hepatic (88–95.6%), renal (4.4–12%)	Metabolism (~75%), renal (~25%)
Hepatic enzymes, f_m_ in %	UGT1A1 (66.8%), UGT1A9 (33.2%)	UGT1A1 (51%), CYP3A4 (21%) ^4^	UGT2B7 (42%), UGT2B15 (58%)	UGT1A9 (13%), UGT2B15 (87%)	UGT2B7 (100%)	UGT1A1 (100)	UGT2B7 (85%), CYP3A4 (15%)
Simulated Fg	85.1%	68–84% (dose dependent)	95% ^3^	96% ^3^	17%	51–52%	93% ^3^
Simulated Fh	99.82%	99%	96%	94%	6%	79%	53%

^1^ CL = clearance, Fg = fraction of drug escaping first-pass intestinal metabolism, Fh = hepatic availability, fu,p = fraction unbound in plasma, MW = molecular weight, Rbp = blood-to-plasma ratio, f_m_ = fraction of metabolism mediated by specific enzyme, Sim+ = Simulations Plus, and Vmax = enzyme maximum rate of metabolism. ^2^ Dolutegravir value used because the cabotegravir (analogue of dolutegravir) value was not available. ^3^ Values are for final adjusted model with empirical scaling factor of 0.25 applied to Vmax values for intestinal metabolism. ^4^ Also minor contributions of UGT1A3, UGT1A9, and oxidative products [63].

**Table 3:** Effects of atazanavir on raltegravir PK in clinical DDI studies ^1^.

Study	Atazanavir Dose, mg	Raltegravir Dose, mg	Observed R_Cmax_	Observed R_AUC_	Simulated R_Cmax_	Simulated R_AUC_
Iwamoto et al. [86]	400 QD	100	1.53	1.72	1.8–1.9	2.0–2.1
Neely et al. [87] ^2^	0/400 QD ^2^	400 BID/400 QD ^2^	1.32	1.37	1.8	1.7–1.8
Zhu et al. [85]	300 BID	400 BID	1.54	1.54	1.8–1.9	1.8–2.0

^1^ BID = twice daily, DDI = drug–drug interaction, QD = once daily, PK = pharmacokinetics, R_Cmax_ = ratio of the Cmax value with atazanavir co-administered to the value without atazanavir co-administered, and R_AUC_ = ratio of the AUC value with atazanavir co-administered to the value without atazanavir co-administered. ^2^ The study compared raltegravir exposures when administered as a single agent at a dose of 400 mg BID compared to a dose of 400 mg QD when co-administered with atazanavir at a dose of 400 mg QD.

**Table 4:** Summary of F, Fa, Fg, and Fh Values for UGT Substrates in Humans ^1^.

Name	F	Fa	Fg	Fh	UGTs Involved	Intestinal Metabolism? ^2^	Reference
Canagliflozin	0.65	1 ^3^	0.75 ^4^	0.86	UGT1A9, UGT2B4	No	Devineni et al. [94]
Dapagliflozin	0.78	1 ^3^	0.90	0.86	UGT1A9, UGT2B7	Yes	Boulton et al. [97]
Diclofenac	0.54	1 ^3^	0.64	0.85	UGT2B7, UGT1A9, UGT1A6, UGT2B15	Yes	Varma et al. [90]
Ertugliflozin	1.05	1.11	1.08	0.88	UGT1A9, UGT2B4, UGT2B7	Yes	Raje et al. [98]
Gemfibrozil	0.98	1 ^3^	1.09	0.90	UGT2B7	Yes	Nishimuta et al. [20]
Lorazepam	0.93	1 ^3^	0.97	0.96	UGT2B4, UGT2B7, UGT2B15	Yes	Varma et al. [90]
Raloxifene	0.02	0.63	0.054	0.593	UGT1A1, UGT1A8, UGT1A9, UGT1A10 ^5^	Yes	Mizuma [12]
Troglitazone	0.45	1 ^3^	0.56	0.80	UGT1A1, UGT1A10	Yes	Nishimuta et al. [20]
Lorazepam	0.93	1 ^3^	0.97	0.96	UGT2B4, UGT2B7, UGT2B15	Yes	Varma et al. [90]
Telmisartan	0.43	0.90	0.75	0.64	UGT1A1, UGT1A3, UGT1A9	Yes	Varma et al. [90]
Oxazepam	0.93	0.93	1.01	0.99	UGT1A9, UGT2B7, UGT2B15	Yes	Varma et al. [90]

^1^ Numbers were either taken as reported or calculated using the approach of Kharasch et al. [89], reported Fa (or assumed 1 in the absence of a reported value), and a liver blood flow rate of 1.5 L/min [99]. F = bioavailability, Fa = fraction absorbed, FDp = fraction of dose passing into the portal vein, Fg = fraction of drug escaping first-pass intestinal metabolism, and Fh = hepatic availability. ^2^ Based on UGT expression in intestine from Basit et al. [8]. ^3^ Fa was assumed to be 1. In this case the Fg actually represented FDp (= Fa × Fg). ^4^ Canagliflozin Fg is most likely underestimated, since UGT1A9 and UGT2B4 are not expressed in the intestines. ^5^ For raloxifene, SULT1E1 contributes to metabolism, but intestinal metabolism is thought to be mainly due to glucuronidation by UGT1A1, 1A8, and 1A10 [100].

**Table 5:** In Silico Substrate Classification for Example UGT Substrates ^1^.

Substrate of ^2^:	UGT1A1	UGT1A3	UGT1A4	UGT1A6	UGT1A8	UGT1A9	UGT1A10	UGT2B7	UGT2B15
Overall Accuracy	85%	85%	88%	85%	87%	88%	85%	84%	91%
**Cabotegravir (UGT1A1, UGT1A9)**	Yes (96%)	No (78%)	No (99%)	No (97%)	Yes	Yes (88%)	Yes (56%)	Yes (80%)	Yes (52%)
**Canagliflozin (UGT1A9, UGT2B4)**	No (50%)	No (66%)	Yes (89%)	No (83%)	No (98%)	No (96%)	No (75%)	No (70%)	No (92%)
**Dolutegravir (UGT1A1)**	Yes (96%)	No (78%)	No (99%)	No (97%)	Yes	Yes (62%)	Yes (56%)	Yes (80%)	Yes (58%)
**Lorazepam (UGT2B7, UGT2B15)**	No (95%)	No (92%)	No (66%)	No (97%)	Yes (81%)	No (96%)	Yes (71%)	Yes (80%)	No (82%)
**Naloxone (UGT2B7)**	No (99%)	Yes (53%)	No (99%)	No (97%)	Yes (72%)	No (96%)	No (97%)	Yes (93%)	No (98%)
**Oxazepam (UGT1A9, UGT2B15)**	No (97%)	No (92%)	Yes (45%)	No (88%)	No (63%)	No (96%)	Yes (64%)	Yes (93%)	No (98%)
**Raltegravir (UGT1A1)**	Yes (90%)	No (49%)	No (99%)	No (91%)	Yes (75%)	Yes (73%)	No (70%)	Yes (74%)	Yes
**Tapentadol (UGT1A9, UGT2B7)**	No (99%)	Yes (90%)	Yes (59%)	No (97%)	Yes (78%)	Yes (97%)	No (90%)	Yes (85%)	No (67%)
**Zidovudine (UGT2B7)**	No (98%)	No (98%)	No (95%)	No (97%)	No	No (69%)	No (97%)	Yes (66%)	No (84%)

^1^ Calculations were done for each of the drugs in the far-left column, with the UGTs thought to metabolize them shown in (). “Yes” or “No” indicates whether the drug is predicted to be a substrate of each enzyme, and “(% confidence)” indicates how likely it is that the classification model is accurate, according to the algorithm. The results are outlined with a red colored square for false positives, a green background for true positives, a tan colored background for false negatives, and a red “Yes/No” for molecules that were out of scope for that prediction (for which confidence was not determined). ^2^ Calculation addresses the question of whether the drugs are substrates of these UGTs (Yes/No), and % confidence that answer is correct.

## Data Availability

Not applicable.
